# Observation on the clinical effect of thunder-fire moxibustion combined with acupressure on ocular muscle spasm: A clinical randomized controlled trial

**DOI:** 10.1097/MD.0000000000021586

**Published:** 2020-08-14

**Authors:** Yi-fei Cao, Tie-jun Li, Yong-mei Xu, Yi Zhang, Jia-yun Nian, Qiang Li, Qiu-yan Ma, Yu Liu, Ying Li, Yan Wu, Chao Yang, Peng-li Cui, Yu-yan Lü, Ying-xin Yang, Yu-hong Zheng

**Affiliations:** a Beijing Hospital of Traditional Chinese Medicine, Capital Medical University; b Shunyi Hospital of Beijing Hospital of Traditional Chinese Medicine, Beijng, China.

**Keywords:** ocular muscle spasm, randomized controlled trial, thunder-fire moxibustion, Traditional Chinese medicine nursing

## Abstract

**Introduction:**

With the rapid development of social economy, peoples dependence on computers and mobile phones is increasing day by day. This causes people to often overuse. Therefore, the incidence of Ocular muscle spasm has been increasing year by year in recent years. The disease usually starts and hides, which seriously affects the patients social image, daily life, and work.

**Methods/design:**

We will compare the clinical efficacy of thunder-fire moxibustion combined with acupressure with pure thunder-fire moxibustion on Ocular muscle spasm using random control method.

**Discussion:**

We aim to find a simple, safe, simple and effective Chinese medicine nursing technology that relieves Ocular muscle spasm.

**Trial registration:**

ClinicalTrials.gov,ChiCTR2000034187, Registered on 27 June 2020.

## 1 Introduction

Ocular muscle spasm (OMS) refers to the involuntary beating of the muscles around the eyes. It can develop from pure upper or lower eyelid spasm to twitching of the upper and lower eyelids, and even to involuntary twitching of muscles on the same side.^[1]^ With the rapid development of social economy, peoples dependence on computers and mobile phones is increasing day by day. This causes people to often overuse. Therefore, the incidence of OMS has been increasing year by year in recent years.^[2,3]^ The disease usually starts and hides, which seriously affects the patients social image, daily life, and work. If it is not treated in time, it will gradually develop to other muscles of the face and even the convulsions of the mouth and mouth, and further develop into facial muscle spasm, which will cause great pain to the patient.^[4]^ At present, the etiology and pathogenesis of the disease are still unclear, and most of them start in adulthood. Bilateral onset is more common at the same time, about 20% of patients are unilateral onset. But as the disease progresses, both eyelids will be involved. The main treatment methods of OMS are drug sedation, acupuncture, and surgery.^[5]^ At present, there are many treatments for this disease, but it is only symptomatic treatment, which cannot effectively solve the essential problems of OMS patients. Oral drugs used to treat OMS in Western medicine often have large side effects and are relatively expensive.^[6]^ At the same time, Western medicine treatment is an invasive operation, increasing the infection rate. Acupuncture and moxibustion is one of the suitable techniques of Traditional Chinese medicine (TCM). Acupuncture has a unique effect on the treatment of this disease. According to the theory of internal organs and meridians of TCM, there is a certain organic connection between the physiological and pathological reactions of the human body and the corresponding acupoints. Stimulation (local compression, acupuncture, heating, acupuncture point closure, etc.) corresponding to acupuncture points can directly affect the patients physiological and pathological reaction process, which can lead to the occurrence or termination of physiological and pathological reactions.^[7]^Acupressure has the effect of warming meridians, while thunder-fire moxibustion has the functions of warming qi and blood, activating meridians and collaterals, which can effectively improve eye blood circulation and promote the normal state of the body.^[8]^ Thrunder-fire moxibustion combined with acupuncture point nursing technology can better relax the muscle tissue of the affected area, regulate nerves, improve local blood circulation, and promote metabolism. This method of operation is easy to operate, has little adverse reactions, a low chance of infection, and is easy for patients to accept. In addition, in the process of clinical treatment, patients should pay attention to rest, avoid excessive use of eyes, fatigue caused by long-term viewing of mobile phones, computers, etc., and avoid external mental stimulation. This study intends to compare the clinical efficacy of thunder-fire moxibustion combined with acupressure with simple thunder-fire moxibustion on OMS by using a randomized control method. Our aim is to explore the simple, safe, convenient, and effective TCM nursing technology to relieve OMS.

## 2 Methods/design

### 2.1 Study design and settings

**Figure 1:**
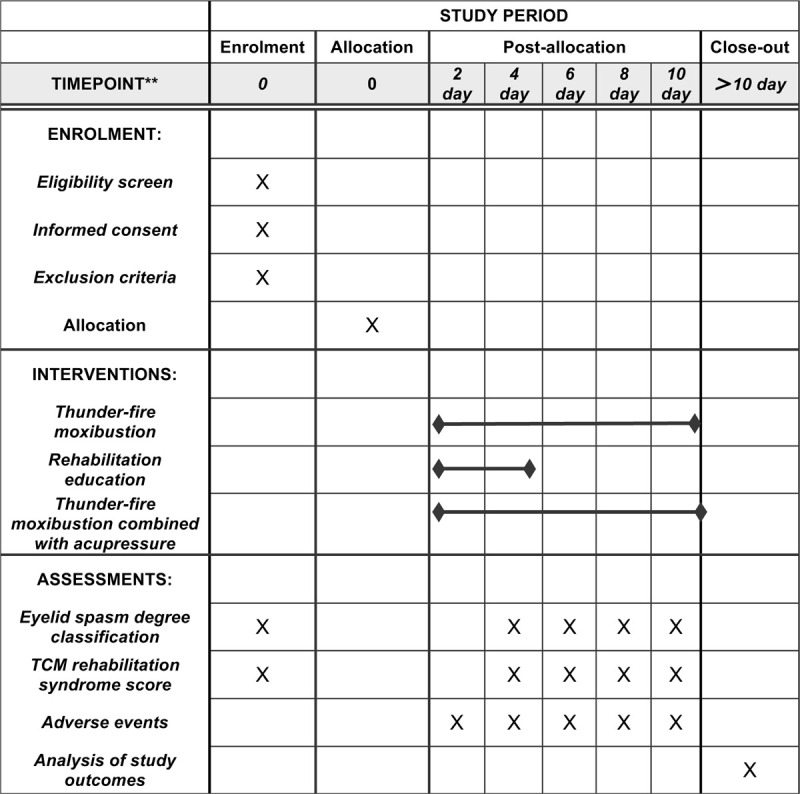
SPIRIT figure for the schedule of enrollment, interventions, and assessments. SPIRIT = Standard Protocol Items: Recommendations for Interventional Trials, TCM = Traditional Chinese medicine.

This study will be a single-blinded, randomized controlled trial with 2 parallel groups. It will be conducted at Beijing Hospital of Traditional Chinese Medicine, Capital Medical University. This protocol was written and based on Standard Protocol Items: Recommendations for Interventional Trials guidelines. If they agree, they will sign an informed consent form. Only those participants who read and agree to the protocol and who sign the informed consent form will take part of the study, following the schedule described in Figure 1.

### 2.2 Ethical aspects

This study will be approved by Beijing Hospital of Traditional Chinese Medicine Ethics Committee. It will be conducted in accordance with the protocol. The rules of confidentiality will be respected. The study protocols is funded through a protocol registry. The study is supported by the TCM evidence-based capacity building project.

### 2.3 Participants

All the cases in this study originated from the ophthalmology clinic and ward of Beijing Hospital of Traditional Chinese Medicine. We will select 76 patients with clinically diagnosed ocular muscle spasm.

#### 2.3.1 Diagnostic criteria

1. Early period is mostly intermittent convulsions of the orbicularis oculi muscles, which can reach other facial muscles on the same side;
2. The degree of convulsions varies, which can be aggravated by factors such as fatigue, nervousness and conversation, and convulsions stop when falling asleep;
3. There were no positive signs on the nervous system.

#### 2.3.2 Inclusion criteria

This study will be conducted in China. We will enroll participants based on the following inclusion criteria:

1. meet the diagnostic criteria of OMS
2. age 18 to 80 years old;
3. have not used relevant therapeutic drugs or have been treated with other drugs, but have been discontinued for more than 2 weeks;
4. with the consent of the patient Later, voluntarily join the research of this subject;

#### 2.3.3 Exclusion criteria

Patients will be excluded if they meet the following criteria:

1. Patients with other neurological diseases;
2. Those under the age of 18 or over 80, pregnant, or lactating women;
3. Combined with patients with serious primary diseases such as heart, cerebrovascular, liver, kidney and hematopoietic system, mental illness;
4. Patients with mental or legal disabilities;
5. Suspect or indeed have a history of alcohol or drug abuse, or other diseases that reduce the possibility of enrollment or complicate enrollment according to the judgment of the investigator, such as frequent changes in the working environment, which are likely to cause loss of follow-up;
6. Those who are known to be allergic to thunder-fire moxibustion components;
7. Patients who are participating in clinical trials of other drugs;

One of the above is excluded.

#### 2.3.4 Criteria for termination of trials

If the following happens, we will terminate the test. Severe acute disease response; severe allergic reactions and other adverse reactions; those who withdraw due to worsening or ineffective eye disease during treatment

### 2.4 Interventions

Control group: Thunder-fire moxibustion. A course of treatment for 10 days, a total of 1 course. Specific operation steps: first turn the thunder-fire moxibustion on the patients forehead, reciprocate, moxibustion 2 to 3 cm from the forehead, and reciprocate left and right for 2 to 3 minutes, until the skin of the patients forehead is reddish; the patient closes his eyes and performs respectively on both eyes Rotate clockwise, the moxa stick is 1 to 2 cm from the acupoint, and each eye is about 2 to 3 minutes. Then moxa sticks from the far and near, respectively, to the points around the eyes of the eyes (*Taiyang, Yuyao, Sibai, Chengqi, Jingming, Cuanzhu apoint,* etc.). Stay for 1 to 2 minutes, and then move to the next acupoint. During the operation, it is necessary to pay attention to the reddish skin around the eyes of the patient. Avoid long time, otherwise it will cause dry eyes.

Intervention group: This group will be treated locally with thunder-fire moxibustion combined with acupressure. A course of treatment for 10 days, a total of 1 course. ① Operator: Having good communication skills after training in relevant professional knowledge. ② Acupoint pressing: *Jingming Point*: locate in the depression above the inner canthus. *Cuanzhu Point*: Located on the face, sunken brows. *Yuyao Point*: Positioned on the forehead with the pupil straight up. *Sizhukong Point*: Positioned at the depression of the brow tip. *Sibai Point*: It is located on the cheek, in the depression of the infraorbital hole. *Taiyang Point*: Locate between the brow tip and the outer canthus, about a depression pointing backwards. *Chengqi Point*: Located on the cheek, between the eyeball and the infraorbital margin. ③ Acupoint compression method: The operator presses with the thumb and abdomen, the direction is vertically downward, and the pressure is gradually increased from light to heavy. It is limited to the local sense of acid, numbness, swelling, heat and the patient can tolerate it. The operator should avoid sudden force application to prevent tissue damage. ④ Compression time: Press after moxibustion, 2 minutes per point. ⑤ Pressing time per point: 5 seconds, pause for 1 second. ⑥ Eye opening and closing state: eyes closed.

### 2.5 Outcome measures

#### 2.5.1 Primary outcome measures

We will formulate standards for evaluating the efficacy of OMS in accordance with the Standards for Diagnosis and Treatment of Traditional Chinese Medical Diseases. The specific evaluation criteria for the efficacy are as follows:

1. Cure: the twitching of the eye cells or facial muscles disappears without recurrence within half a year;
2. Effective: the spasm level of the eye cells or facial muscles is reduced, the number of twitching is reduced, and the time between twitching is shortened;
3. Ineffective: The degree of spasm of the ocular muscles has not improved. All patients were graded according to the degree of blepharospasm. Level 0 is without spasm. Level 1 is an increase in convulsions caused by external stimuli. Grade 2 is mild spasm, slight twitching of eyelids, and no dysfunction. Grade 3 is moderate spasm, with obvious eyelid spasm and mild dysfunction. Grade 4 is severe spasm, severe blepharospasm, obvious dysfunction, affecting work and life.

#### 2.5.2 Efficacy evaluation

Refer to “Guidelines for Clinical Research of New Chinese Medicine”. Efficacy was evaluated by the ratio of the difference between the points before and after treatment compared to the points before treatment. Syndrome treatment efficiency = (total points before treatment total points after treatment)/total points before treatment × 100%.

1. Clinical control: clinical symptoms and signs disappeared or basically disappeared, and syndrome scores were reduced by ≥95%;
2. Significant effect: clinical symptoms and signs were significantly improved, and syndrome scores were reduced by 70%;
3. The signs and symptoms have improved, and the syndrome scores have decreased by ≥30%;
4. Ineffective: the clinical symptoms and signs have not improved significantly, and the syndrome scores have decreased by less than 30%.

### 2.6 Sample size calculation

With reference to previous clinical trials on the application of thunder-fire moxibustion combined with acupressure in the treatment of OMS, the standard deviation was 3.13. The purpose of this trial was to observe the difference in relief of ocular muscle spasm between patients with conventional thunder-fire moxibustion and acupressure. We expect that the treatment group can cause 90% of the changes to be clinically meaningful, in line with the superior effect design of the measurement data, and the sample size formula of the measurement index is as follows: 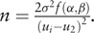. Combined with clinical practice, α = 0.05, β = 0.2, and the table *f* (*α*, *β*) = 7.9 then the calculated sample size is 33 cases in each group. According to the 15% shedding rate, the sample size in each group is 38 cases, and the treatment group and the control group total 76 cases.

### 2.7 Randomization and blinding

The random method will use the closed envelope method for block randomization. Use the SAS software PROC PLAN procedure statement for random grouping. The subjects will be numbered 1 to 76 according to the order in which the patients were enrolled, and random numbers and assignments corresponding to the order of inclusion were obtained. The groups will be sealed in opaque envelopes, and the order of inclusion will be affixed to the surface of the envelopes. Randomization envelopes and distributions are performed by individuals who are not involved in treatment and evaluation.

### 2.8 Statistical analysis

According to the m-ITT principle, all randomized cases will be included. And there is at least 1 treatment and 1 post-baseline data. The missing data are not filled, and the actual observation values are used for statistical analysis. The measurement data are described by mean, standard deviation, median, and quartile. If the data meets the normal distribution, it is compared with the baseline value at the time of enrollment, and a paired T test is used. Differences in efficacy between the 2 groups will be compared using independent sample *t* tests. If the normal distribution is not satisfied, a non-parametric test is used. The count data is described using a composition ratio. If the normal distribution is satisfied, the changes before and after treatment in the 2 groups are tested by X^2^. If the normal distribution is not satisfied, a non-parametric test is used. The statistical software is SPSS 24.0, when *P* < .05, it showed that the difference is statistically significant.

### 2.9 Data management

**Figure 2:**
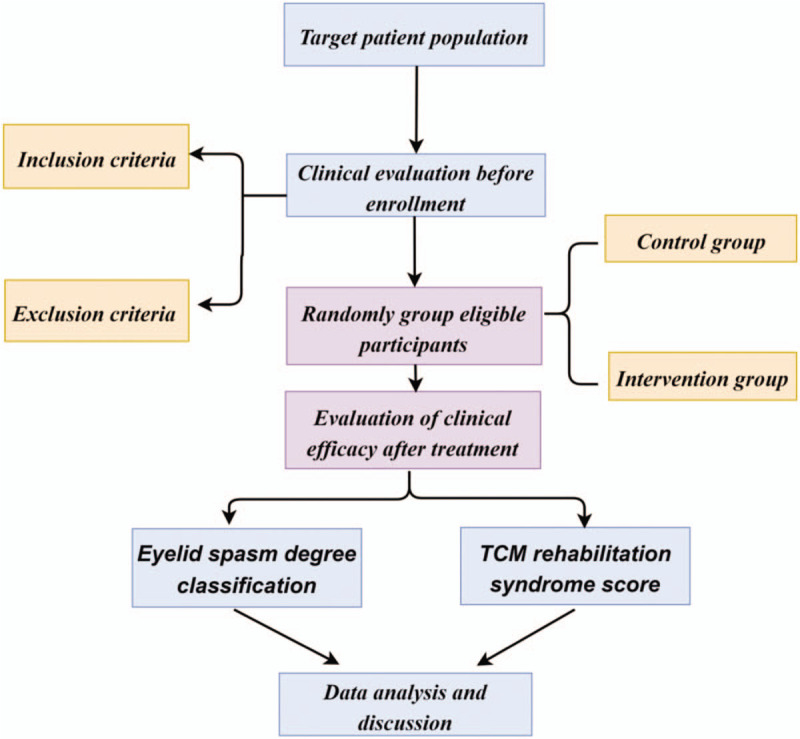
Study design flow chart.

Information obtained from the evaluation of each participant will be recorded on a paper print-out. The information will then be handwritten on a paper document case report form and entered into an Excel file for future statistical analyses. In accordance with the Personal Information Protection Act, the names of all participants will not be disclosed, and a unique identifier number given during the trial will be used to identify participants. All of the participants will be informed that the clinical data obtained in the trial will be stored in a computer and will be handled with confidentiality. The participants written consent will be stored by the principal investigator (Fig. 2).

## 3 Discussion

OMS is an unexplained and involuntary muscle spasm and convulsions in the innervation area of the facial nerve, which is more common in the middle-aged and elderly people, and is more common in the 50 to 70 age group, and more women than men.^[9]^ In the early stage of the patient, the number of blinks increased, and the eyelids became heavy, and paroxysmal opening of the eyes often occurred during gaze. Because the control center of blepharospasm is not yet clear, there are no targeted drugs.^[10]^ At first, blepharospasm was considered a manifestation of mental illness, so antipsychotic drugs were used. It has recently been discovered that the efficacy of these drugs is not due to the treatment of mental illness, but to the motor system. Most patients do not respond partially or completely to medication. The best case is a partial short-term relief. There are individual differences in the response of OMS patients to various drugs, but there is no way to predict which patient will respond to which specific drug. Tricyclic antidepressants do not directly affect blepharospasm, but they are helpful for patients with aggravated symptoms of depression.^[11]^ Various drugs have shown partial efficacy for blepharospasm. The goal of medication is to improve symptoms and reduce complications. Botulinum toxin can improve orbicularis oculi muscle spasm and autonomic symptoms. The efficacy of botulinum toxin injection treatment is generally temporary, requiring repeated treatment for a long time. Common complications of botulinum toxin injection include drooping eyelids and weakness of facial muscles. Common complications of surgical treatment include scars and edema. Resection of the orbicularis oculi muscle is permanent.

At present, TCM nursing technology plays an important role and advantage in preventing health care, promoting rehabilitation, and alleviating pain. Thunder-fire moxibustion belongs to the nursing technology of TCM, which has the function of warming and ventilating.^[12]^ It can dredge the eye blood and warm the meridians through the warming effect; and acupressure is the earliest application of massage therapy, which belongs to the external treatment of TCM category.^[13]^ Acupressure specifically refers to the use of the doctors hands and the use of standardized movement techniques to adjust the bodys physiological functions and pathological changes, to achieve the functions of dredging the meridians, promoting blood circulation and removing blood stasis, adjusting viscera and blood, and improving the bodys ability to resist disease.^[7,14]^ Related literature confirms that the use of thunder-fire moxibustion or acupressure can treat or delay the development of ocular muscle spasm. At present, the clinical application of thunder-fire moxibustion and acupressure nursing technology for the treatment of ocular muscle spasm has irregular operation procedures and inconsistent treatment frequency and time, which may be the reason for affecting the evaluation of efficacy. This study proposes a hypothesis, intends to adopt a randomized controlled clinical research method, standardize the operation steps, and provide evidence-based medical evidence for the improvement of OMS in TCM nursing technology. At the same time, we also hope that this study can provide simple and easy-to-promote nursing technology for the clinical diagnosis and treatment of OMS.

## Acknowledgments

The authors would like to thank all the trial participants. The authors are grateful for the support for this study: trial coordinating team, surgical staff, nurses, and research departments.

## Author contributions

YFC, TJL, YMX, and YZ designed the study protocol. YL, YW, CY, and YYL drafted the manuscript. JYN, QL, and PLC reviewed the study protocol and drafted the manuscript. QYM, YL, and YXY is responsible for the statistical design and analysis as trial statistician. All authors carefully read and approved the final version of the manuscript.
